# New-Onset Postoperative Seizures in Patients With Diffuse Gliomas: A Risk Assessment Analysis

**DOI:** 10.3389/fneur.2021.682535

**Published:** 2021-06-18

**Authors:** Lianwang Li, Guanzhang Li, Shengyu Fang, Kenan Zhang, Ruoyu Huang, Yinyan Wang, Chuanbao Zhang, Yiming Li, Wei Zhang, Zhong Zhang, Qiang Jin, Dabiao Zhou, Xing Fan, Tao Jiang

**Affiliations:** ^1^Beijing Neurosurgical Institute, Capital Medical University, Beijing, China; ^2^Department of Neurosurgery, Beijing Tiantan Hospital, Capital Medical University, Beijing, China; ^3^Research Units of Accurate Diagnosis and Treatment of Brain Tumors and Translational Medicine, Chinese Academy of Medical Sciences, Beijing, China

**Keywords:** diffuse glioma, new-onset postoperative seizures, risk factors, IDH1 mutation, overall survival

## Abstract

**Background:** Glioma-related epilepsy (GRE) is the most common presenting sign of patients with diffuse glioma. According to clinical experience, new-onset postoperative seizures can be observed even in patients without preoperative GRE. The current study mainly aimed to explore the risk factors of new-onset postoperative seizures in those patients. In addition, the prognostic value of new-onset postoperative seizures was also discussed.

**Methods:** Data of 313 patients without GRE were retrospectively reviewed. Chi-square test or Fisher's exact test were first performed to compare categorical variables between patients with new-onset postoperative seizures and those without. Subsequently, binary logistic regression analysis was conduct to further assess risk factors of new-onset postoperative seizures. Kaplan-Meier and Cox analysis were used to investigate the prognostic value of new-onset postoperative seizures for progression-free survival (PFS) and overall survival (OS).

**Results:** Patients with low-grade tumors (*p* = 0.006), isocitrate dehydrogenase 1 (IDH1) mutation (*p* = 0.040) or low Ki-67 expression (*p* = 0.005) showed a higher incidence of new-onset postoperative seizures. IDH1 mutation was identified as the only independent predictor for new-onset postoperative seizures (OR, 2.075; 95% CI, 1.051–4.098; *p* = 0.035). Additionally, new-onset postoperative seizure occurrence was demonstrated as an independent predicter of prolonged OS (OR, 0.574; 95% CI, 0.335–0.983; *p* = 0.043), while younger age, gross total resection, low-grade and IDH1 mutation were independently correlated with prolonged OS and PFS.

**Conclusions:** IDH1 mutation is an independent predictor for new-onset postoperative seizures in patients without preoperative GRE. Moreover, new-onset postoperative seizures can independently predict prolonged OS in those patients. The results of the current study can contribute to improving the individualized management of diffuse glioma.

## Background

Glioma-related epilepsy (GRE) is a common clinical symptom of diffuse gliomas. It can be observed in 65–90% of cases with diffuse low-grade gliomas (WHO grade 2) and 40–64% of cases with diffuse high-grade gliomas (WHO grades 3 and 4), with a clinical presentation varied from focal to tonic-clonic seizures (1). GRE can significantly decrease the patients' quality of life, and bring heavy financial and psychological burdens to them and their families (2).

For a long time, the sole purpose of glioma treatment has been to prolong survival. In recent decades, due to the increasing concern of the patients' quality of life, seizure control has been considered as a secondary goal for glioma treatment (3). Currently, the optimal treatment strategy for GRE is a combination of antiepileptic drugs (AEDs) and antitumor treatments, including surgical resection and adjuvant radiotherapy and/or chemotherapy (1). However, despite the aforementioned treatment strategy, seizure control is still not achieved in over 20% of patients (4). In addition, AED management, which is the crucial part of postoperative antiepileptic therapy, is not in a regular way. For instance, the prophylactic use of AEDs, and the timing of AED reduction/withdrawal are still very controversial between different medical centers. This kind of circumstance is largely due to the lack of compelling clinical summary which can guide individualized antiepileptic therapy, e.g., risk factors related to postoperative seizure control.

Factors associated with postoperative seizure control have been investigated in numerous studies (5, 6). In 2018, we performed a systemic review and meta-analysis basing on relevant studies, and identified that younger age, clinical presentation of focal seizures, a prolonged history of seizures prior to surgery and non-gross total resection (non-GTR) had negative impacts on postoperative seizure control (7). For patients with the above risk factors, aggressive AEDs therapy is potentially helpful for improving seizure control.

To date, several surgical modalities can be applied to achieve potential better seizure outcome in patients with preoperative GRE. For instance, electroencephalogram (EEG) and/or intra-operative electrocorticography can be applied to facilitate the identification of the epileptogenic zone (EZ), and extended resection and/or multiple subpial transection can be used to remove the potential EZ (8). Meanwhile, it's worth noting that postoperative seizures can occur even in some patients without preoperative GRE (9). This subset of patients should be of particular concern. The absence of preoperative seizure history prevents them from receiving antiepileptic surgical procedures and aggressive AED therapy, from which they could have benefit a lot. Accordingly, characterizing this subset of patients is of great significance for the individualized management of diffuse glioma. In the current study, we aimed mainly to explore the risk factors of new-onset postoperative seizures in patients without preoperative GRE, moreover, the association between new-onset postoperative seizures and patient survival was also investigated.

## Methods

### Study Population

Data of patients who underwent surgical resection at Beijing Tiantan Hospital between January 2006 and December 2018 were retrospectively reviewed. The inclusion criteria were as follows: (1) newly diagnosed and histologically confirmed supratentorial diffuse glioma; (2) no history of preoperative seizures; (3) age between 18 and 75 years old. The exclusion criteria were as follows: (1) history of biopsy, radiotherapy and chemotherapy prior to operation; (2) previous malignancy or other concomitant malignant disease; (3) multifocal tumors. Ultimately, a total of 313 patients were enrolled. Demographic, clinical and follow-up data were collected from the Chinese Glioma Genome Atlas (CGGA) database (http://www.cgga.org.cn/). The study was approved by the Ethics Committee of Beijing Tiantan Hospital, and written informed consent was obtained from all the patients or their legal guardians.

### Histopathological Diagnosis and Molecular Markers

Tumor samples obtained from surgical resection were immediately snap-frozen in liquid nitrogen. Subsequently, hematoxylin and eosin-stained sections were prepared to assess the percentage of tumor cells, samples with over 80% tumor cells were selected for further analysis. The histopathological diagnosis was made independently by two experienced neuropathologists. Any inconsistent judgments would be resolved by a third expert. As for molecular markers, isocitrate dehydrogenase 1 (IDH1) mutation was detected by pyrosequencing, while p53 and Ki-67 expression were assessed via immunohistochemistry.

### Evaluation of the Extent of Resection

Pre- and postoperative magnetic resonance images (within 72 h after surgery) were reviewed. The extent of resection (EOR) was stratified as gross total resection (GTR) and non-GTR. For non-enhancing tumors, GTR was defined as no residual high-signal area on postoperative T2-weighted images. For enhancing tumors, GTR was defined as no residual high-signal area on postoperative contrast-enhanced T1-weighted images. The EOR was evaluated independently by two experienced neurosurgeons who were blinded to the clinical outcomes. Cases with inconsistent results would be reviewed by a third neurosurgeon for final decision.

### Follow-Up

All patients received prophylactic AEDs (valproate or levetiracetam) after surgery. Follow-up data were collected by scheduled clinical visits or telephone interviews. Postoperative seizures were defined as at least 1 unprovoked epileptic seizure from the day of out-discharge. The collected information included postoperative seizure occurrence, adjuvant therapy (radiotherapy and/or chemotherapy), AEDs regimen, progression-free survival (PFS) and overall survival (OS). PFS was defined as the time from surgery to radiographic progression (identified according to the Response Assessment in Neuro-Oncology criteria), and OS was defined as the time from surgery until death.

### Statistical Analysis

SPSS software (version 16.0, SPSS Inc., Chicago, IL, USA) were used for data management and statistical analysis. Chi-square test or Fisher's exact test were used for comparison of categorical variables between patients with new-onset postoperative seizures and those without. Binary logistic regression analysis with backward stepwise selection was performed to identify independent risk factors associated with new-onset postoperative seizures. For survival analysis, Kaplan-Meier method (log-rank test) was applied to investigate the correlation of new-onset postoperative seizures with PFS and OS. The R project for statistical computing (version 4.0.0, available at www.r-project.org) was used for graphic plotting (survival curves and nomograms) with packages including “survival,” “ggplot2,” and “rms.” At last, backward stepwise Cox regression analysis was used to further evaluate the prognostic value of new-onset postoperative seizures. A *p* < 0.05 was considered statistically significant. Odds ratio (OR) and 95% confidence interval (CI) were used to evaluate the correlations and measure relevant results.

## Results

### Patient Characteristics

Among the enrolled 313 patients without preoperative GRE, 96 patients experienced new-onset postoperative seizures, while the other 217 did not. Clinical characteristics of enrolled patients are summarized in Table 1. Low-grade gliomas (*p* = 0.006), IDH1 mutation (*p* = 0.040) and low Ki-67 expression (defined as a Ki-67 index <10%, *p* = 0.005) were identified to be associated with a higher incidence of new-onset postoperative seizures. No significant difference was detected in age, sex, tumor side, temporal lobe involvement, preoperative functional deficits, EOR, p53 expression, postoperative adjuvant therapy between patients with new-onset postoperative seizures and those without.

**Table 1:** Clinical characteristics of enrolled patients.

**Variables**	**Total**	**New-onset seizures**	**No seizures**	***P*-value**
No. of patients	313	96	217	
Sex				0.625
Male	176	52	124	
Female	137	44	93	
Age				
<45 years	163	54	109	0.326
≥45 years	150	42	108	
Side				0.835
Left	136	41	95	
Right	155	47	108	
Bilateral	22	8	14	
Location				0.231
Temporal lobe	131	45	86	
Others	182	51	131	
Functional deficits				0.097
Yes	126	32	94	
No	187	64	123	
EOR				0.777
GTR	157	47	110	
Non-GTR	156	49	107	
WHO grade				0.006^*^
Low-grade	149	57	92	
High-grade	164	39	125	
IDH1 mutation				0.040^*^
Yes	165	57	108	
No	109	25	84	
p53 expression				0.293
Over-expressed	150	40	110	
Not	107	35	72	
Ki-67 expression				0.005^*^
Low	116	46	70	
High	108	24	84	
Combined radiochemotherapy				0.260
Yes	151	55	96	
No	136	41	95	

*EOR, extent of resection; GTR, gross total resection; IDH1, isocitrate dehydrogenase 1*.

* *P < 0.05 was considered as statistically significant*.

### Risk Factors for New-Onset Postoperative Seizures

**Figure 1:**
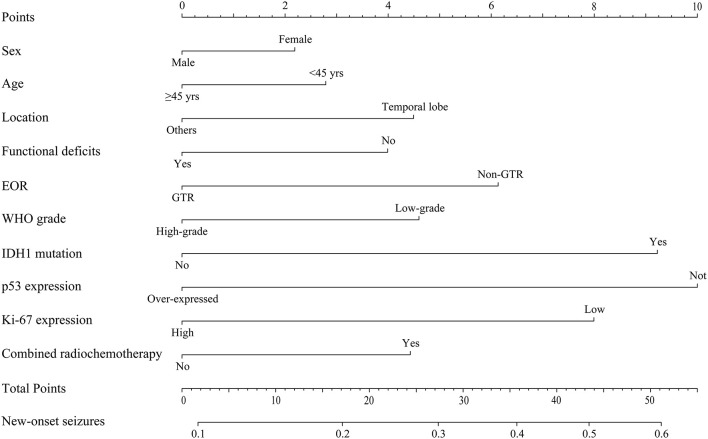
Predictive nomogram for new-onset postoperative seizures. The nomogram showed risk factors of new-onset seizure in patients with diffuse gliomas. EOR, extent of resection; WHO, World Health Organization.

Multivariate binary logistic regression analysis was applied to determine risk factors for new-onset postoperative seizures. All relevant ordinal categorical variables were incorporated into the regression model. Due to incomplete data, only 190 patients were analyzed (Supplementary Table 1). Only IDH1 mutation was identified as an independent predictor for new-onset postoperative seizures (OR, 2.075; 95% CI, 1.051–4.098; *p* = 0.035). A nomogram for new-onset postoperative seizure occurrence is shown in Figure 1.

### Prognostic Value of New-Onset Postoperative Seizure in Diffuse Gliomas

**Figure 2:**
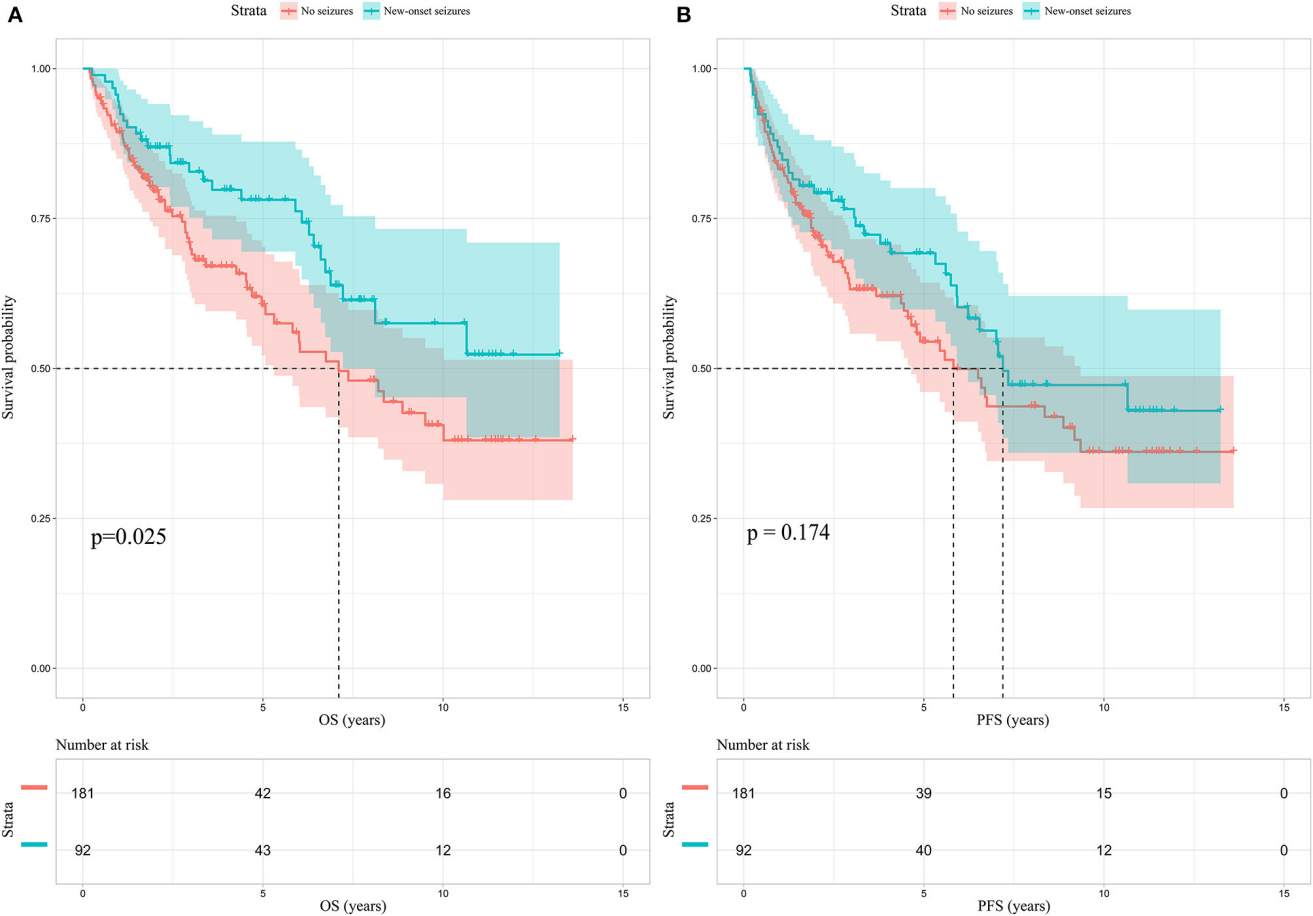
Kaplan-Meier survival analysis in patients without preoperative glioma-related seizures. **(A)** New-onset postoperative seizures can predict prolonged OS (*p* = 0.025, log-rank test); **(B)** no significant difference is identified in PFS between patients with new-onset postoperative seizures and those without. OS, overall survival; PFS, progression-free survival.

**Figure 3:**
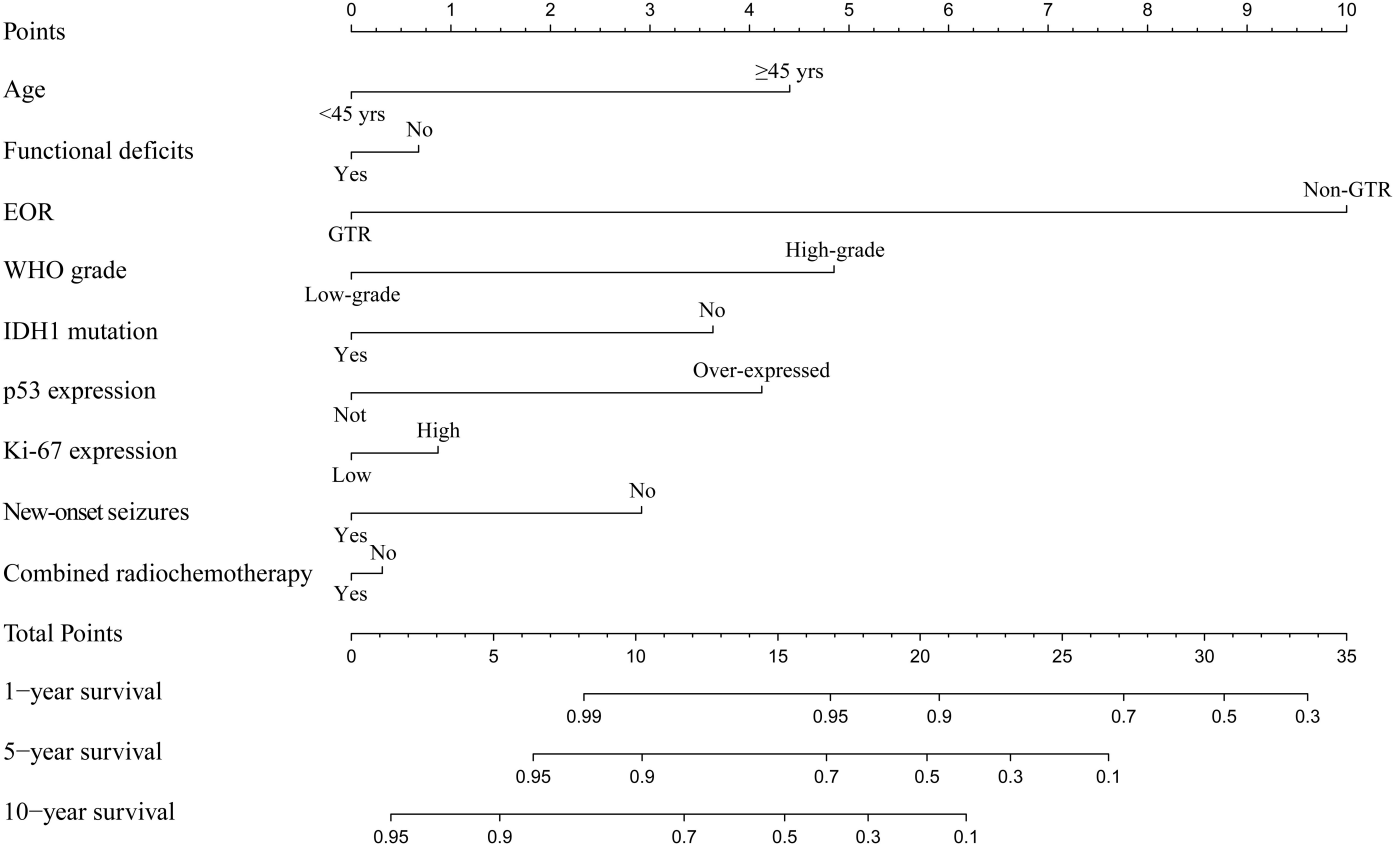
Prognostic nomogram for survival outcome. The nomogram shows risk factors correlate with the overall survival of patients without preoperative seizures. EOR, extent of resection; WHO, World Health Organization.

Among the enrolled 313 patients, 40 patients were lost to follow-up, the follow-up rate was 87.22%. The mean follow-up time was 55.33 months (range 5.73–165.43 months). Kaplan-Meier analysis was performed to investigate the prognostic value of new-onset postoperative seizures. The result showed that patients with postoperative new-onset postoperative seizures experienced longer OS compared with those without (*p* = 0.025, log-rank test, Figure 2A). In contrast, no significant difference was detected in PFS between the two groups (*p* = 0.174, log-rank test, Figure 2B). Subsequently, univariate and multivariate Cox proportional hazard regression analysis was performed to further explore the prognostic value of new-onset postoperative seizures. One hundred and seventy-one patients with available complete data were incorporated into the model. The results are shown in Table 2. New-onset postoperative seizures was identified as an independent predicter of prolonged OS (OR, 0.574; 95% CI, 0.335–0.983; *p* = 0.043). In addition to new-onset postoperative seizures, younger age (*p* = 0.002), GTR (*p* < 0.001), low-grade (*p* < 0.001), IDH1 mutation (*p* = 0.007) and low p53 expression (*p* = 0.005) were shown to be independently associated with prolonged OS. A prognostic nomogram for OS is presented in Figure 3. As for PFS, younger age (*p* = 0.009), GTR (*p* < 0.001), low-grade (*p* = 0.001), IDH mutation (*p* = 0.004) and low p53 expression (*p* = 0.001) were identified as independent positive predictors.

**Table 2:** Univariate and multivariate COX analysis of risk factors associated with survival in patients with diffuse gliomas.

**Variable**	**OS**				**PFS**			
	**Univariable**	**Multivariable**			**Univariable**	**Multivariable**		
	***P***	**HR**	**95% CI**	***P***	***P***	**HR**	**95% CI**	***P***
Male	0.934				0.963			
Age ≥45 years	<0.001^*^	2.266	1.348–3.810	0.002	<0.001^*^	1.935	1.178–3.178	0.009^*^
Temporal lobe	0.205				0.094			
Functional deficits	0.004^*^				0.004^*^			
GTR	<0.001^*^	0.159	0.090–0.280	<0.001^*^	<0.001^*^	0.218	0.132–0.361	<0.001^*^
High WHO grade	<0.001^*^	2.616	1.541–4.442	<0.001^*^	<0.001^*^	2.300	1.420–3.725	0.001^*^
IDH1 Mutation	<0.001^*^	0.482	0.283–0.823	0.007^*^	<0.001^*^	0.472	0.283–0.786	0.004^*^
p53 over-expression	0.016^*^	2.209	1.272–3.838	0.005^*^	0.006^*^	2.338	1.397–3.913	0.001^*^
Ki-67 high expression	0.002^*^				0.001^*^			
New-onset seizures	0.026^*^	0.574	0.335–0.983	0.043^*^	0.175			
Combined radiochemotherapy	0.002^*^				<0.001^*^			

*OS, overall survival; PFS, progression-free survival; GTR, gross total resection; IDH1, isocitrate dehydrogenase 1; OR, odds ratio; CI, confidence interval*.

* *P < 0.05 was considered to be statistically significant*.

## Discussion

In terms of postoperative management for diffuse glioma, there are many controversial issues that need to be clarified. The use of prophylactic AEDs in patients without preoperative GRE is definitely one of them (1). There is a plenty of evidences to prove that AED prophylaxis do not significantly reduce the incidence of postoperative seizures in brain tumor patients without preoperative seizures (10, 11). Accordingly, Many experts believe that routine prophylactic AED use has no benefit for this group of patients (12). However, in our opinion, AED prophylaxis is not meaningless for patients without preoperative GRE, as new-onset postoperative seizures can indeed be observed in some of them. A more plausible explanation is that for one subset of patients, routine AED prophylaxis is unnecessary or at least sufficient for postoperative seizure control; while for the other subset of patients, routine AED prophylaxis is insufficient and should be more aggressive. In consequence, the identification of markers for distinguishing between the two subsets is of great importance.

In the current study, we explored the risk factors of new-onset postoperative seizures in patients without preoperative GRE, and identified that IDH1 mutation was the only independent predictor for new-onset postoperative seizures by multivariate analysis. The result suggested that for patients with IDH1 mutant gliomas, more attention and closer monitoring for seizure should be given during postoperative follow-up even if they did not have preoperative GRE, and long-term AED prophylaxis is potential helpful. IDH1 mutation is considered as an early event in glioma-genesis, and has gained major importance in the 2016 WHO classification of diffuse gliomas (13). In addition, the close correlation of IDH1 mutation with preoperative GRE has already been confirmed (14). The accumulation of D-2-hydroxyglutarate (D2HG), which is structurally similar to the major excitatory neurotransmitter glutamate, is supposed to be a major mechanism behind such correlation. As an important metabolic enzyme, IDH1 can catalyze the oxidative decarboxylation of isocitrate to a-ketoglutarate. While mutant IDH1 can reduce a-ketoglutarate to D2HG, which results in the accumulation of D2HG in tumor microenvironment. The accumulation of D2HG can lead to overexcitation of neurons and thus induce epilepsy (15, 16). This mechanism can also explain the association between IDH1 mutation and new-onset postoperative seizures. Generally, we evaluate the EOR basing on tumor boundary seen on neuroimaging. However, a radiologically proven GTR does not mean that all tumor cells have been removed, and the presence of glioma cells outside the radiological tumor borders has been demonstrated (17). The remaining IDH1 mutant cells can still affect the surrounding microenvironment, and lead to new-onset seizures. Overall, in order to reduce the incidence of postoperative GRE, a GTR basing on the “epileptogenic boundary,” which is actually the histological boundary, can be more effective.

Another potential mechanism behind the correlation of IDH1 mutation with new-onset postoperative seizures is related to seizure spread network. It is reported that patients with IDH1 mutant gliomas have higher global functional connectivity, which is difficult to recover even after the tumor is removed (18, 19). In contrast, gliomas carrying wild-type IDH1 are often more aggressive and thus more likely to cause destruction of brain networks, which may add barriers to the spread of epileptic discharges (20).

We also found that WHO grade and Ki-67 expression were negatively associated with new-onset postoperative seizures. It has been widely accepted that patients with low-grade gliomas have a higher incidence of preoperative GRE (1). In the current study, we identified that low-grade glioma patients without preoperative GRE also have a higher incidence of new-onset postoperative seizures. IDH1 mutant status may be one of the decisive factors behind such relationship, because IDH1 mutation is frequently observed in diffuse gliomas but rarely seen in primary glioblastomas (WHO grade IV) (20). Maybe that was also why WHO grade showed no statistical significance in regression analysis. As for Ki-67, it is an effective biomarker to determine the proliferative activity of tumors (21). As mentioned above, tumors with stronger proliferation ability are more likely to cause damage to brain networks, and impede the spread of epileptic discharges. Therefore, it's not difficult to understand the association between low Ki-67 expression and new-onset postoperative seizures. The two factors above can be also used as references for close observation and long-term AED prophylaxis.

The prognostic value of new-onset postoperative seizures was also investigated. In 2018, we performed a meta-analysis to determine the association between preoperative GRE and clinical outcomes, and identified that GRE at presentation is significantly correlated with prolonged OS in patients with diffuse glioma (14). Consistent with this previous study, here we also demonstrated that new-onset postoperative seizures could predict longer OS in patients without preoperative GRE. Given the correlation of new-onset postoperative seizures with the above proven positive prognostic factors, like lower WHO grade and IDH1 mutation, such result was predictable (22, 23). However, early postoperative seizures do not seem to predict a good prognosis. Dewan et al. reported that glioma patients with early postoperative seizures have a significant shorter median survival (3 months) than those without (15.6 months) (24). The worse prognosis of patients with early postoperative seizures may due to the hemorrhage and severe intracranial hypertension following epilepsy (25). For patients with early postoperative seizures, corresponding active examinations and treatments should be proceed in a timely manner.

The study has several limitations. For instance, as a proportion of patients were followed up by telephone interviews, EEG was not applied for the definite diagnosis of seizures in those patients. Moreover, the study was limited by its retrospective nature, the results need to be verified in a prospective set of patients. Efforts to further explore risk factors of new-onset postoperative seizures, and reduce the incidence of postoperative seizures through patient-tailored antiepileptic therapies should remain the priorities of future studies.

## Conclusions

The current study identified that IDH1 mutation was an independent risk factor for new-onset postoperative seizures in patients without preoperative GRE. Moreover, new-onset postoperative seizure occurrence is demonstrated as an independent predictor of prolonged OS in those patients. To the best of our knowledge, this is the first study to investigate the above topics in patients with diffuse glioma. The results can provide new evidences for improving the management of diffuse glioma, and benefit relevant patients from the application of tailored antiepileptic therapies.

## Data Availability Statement

Publicly available datasets were analyzed in this study. This data can be found here: Chinese Glioma Genome Atlas (CGGA) database (http://www.cgga.org.cn/). The other raw data supporting the conclusions of this article will be made available by the authors, without undue reservation.

## Ethics Statement

The studies involving human participants were reviewed and approved by the Ethics Committee of Beijing Tiantan Hospital. The patients/participants provided their written informed consent to participate in this study.

## Author Contributions

LL and XF: study concept and design. LL, GL, KZ, and SF: data acquisition and analysis. LL and RH: formal analysis and investigation. LL: writing—original draft preparation. XF and YL: writing—review and editing. YW and TJ: funding acquisition. CZ, WZ, ZZ, QJ, and DZ: resources. XF and TJ: supervision. All authors contributed to the article and approved the submitted version.

## Conflict of Interest

The authors declare that the research was conducted in the absence of any commercial or financial relationships that could be construed as a potential conflict of interest.

## Abbreviations

GRE: glioma-related epilepsy
PFS: progression-free survival
OS: overall survival
IDH1: isocitrate dehydrogenase 1
AEDs: antiepileptic drugs
EOR: extent of resection
GTR: gross total resection
EEG: electroencephalogram
EZ: epileptogenic zone
CGGA: Chinese Glioma Genome Atlas.
