# Supplementary material for: New-Onset Postoperative Seizures in Patients With Diffuse Gliomas: A Risk Assessment Analysis
Source: Front Neurol. 2021 Jun 18;12:682535. doi: 10.3389/fneur.2021.682535 (PMC8250134; doi:10.3389/fneur.2021.682535)
Supplement: Supplementary file 1 [file Table_1.docx]

**Table S1** Backward stepwise logistic regression analysis with potential variables predicting new-onset postoperative seizures.

|  | | p-value | OR | 95% CI | |
| --- | --- | --- | --- | --- | --- |
|  |  |  |  | Lower | Upper |
| Step 1 | Male | 0.723 | 0.891 | 0.471 | 1.686 |
|  | Age≥45 yrs | 0.684 | 0.863 | 0.425 | 1.754 |
|  | Functional deficits | 0.542 | 0.810 | 0.412 | 1.593 |
|  | Temporal lobe involvement | 0.482 | 1.267 | 0.655 | 2.450 |
|  | GTR | 0.324 | 0.724 | 0.381 | 1.376 |
|  | High WHO grade | 0.599 | 0.785 | 0.318 | 1.938 |
|  | IDH1 Mutation | 0.253 | 1.625 | 0.707 | 3.734 |
|  | p53 over-expression | 0.113 | 0.591 | 0.308 | 1.132 |
|  | Ki-67 high expression | 0.343 | 0.657 | 0.275 | 1.568 |
|  | Combined radiochemotherapy | 0.526 | 1.263 | 0.614 | 2.595 |
|  | Constant | 0.617 | 0.739 |  |  |
| Step 2 | Age≥45 yrs | 0.696 | 0.869 | 0.428 | 1.762 |
|  | Functional deficits | 0.546 | 0.812 | 0.413 | 1.596 |
|  | Temporal lobe involvement | 0.485 | 1.265 | 0.654 | 2.447 |
|  | GTR | 0.317 | 0.721 | 0.379 | 1.369 |
|  | High WHO grade | 0.599 | 0.784 | 0.318 | 1.938 |
|  | IDH1 Mutation | 0.259 | 1.611 | 0.704 | 3.690 |
|  | p53 over-expression | 0.107 | 0.587 | 0.306 | 1.123 |
|  | Ki-67 high expression | 0.341 | 0.655 | 0.274 | 1.564 |
|  | Combined radiochemotherapy | 0.523 | 1.265 | 0.615 | 2.602 |
|  | Constant | 0.537 | 0.698 |  |  |
| Step 3 | Functional deficits | 0.501 | 0.795 | 0.408 | 1.550 |
|  | Temporal lobe involvement | 0.455 | 1.284 | 0.667 | 2.473 |
|  | GTR | 0.332 | 0.729 | 0.385 | 1.381 |
|  | High WHO grade | 0.579 | 0.774 | 0.314 | 1.909 |
|  | IDH1 Mutation | 0.196 | 1.689 | 0.763 | 3.741 |
|  | p53 over-expression | 0.113 | 0.593 | 0.311 | 1.132 |
|  | Ki-67 high expression | 0.338 | 0.653 | 0.273 | 1.562 |
|  | Combined radiochemotherapy | 0.529 | 1.260 | 0.614 | 2.586 |
|  | Constant | 0.394 | 0.636 |  |  |
| Step 4 | Functional deficits | 0.486 | 0.789 | 0.405 | 1.536 |
|  | Temporal lobe involvement | 0.470 | 1.273 | 0.662 | 2.448 |
|  | GTR | 0.343 | 0.735 | 0.389 | 1.390 |
|  | IDH1 Mutation | 0.137 | 1.791 | 0.832 | 3.860 |
|  | p53 over-expression | 0.103 | 0.585 | 0.307 | 1.114 |
|  | Ki-67 high expression | 0.158 | 0.578 | 0.270 | 1.238 |
|  | Combined radiochemotherapy | 0.628 | 1.185 | 0.597 | 2.349 |
|  | Constant | 0.318 | 0.596 |  |  |
| Step 5 | Functional deficits | 0.493 | 0.792 | 0.407 | 1.541 |
|  | Temporal lobe involvement | 0.478 | 1.267 | 0.659 | 2.436 |
|  | GTR | 0.322 | 0.726 | 0.384 | 1.369 |
|  | IDH1 Mutation | 0.131 | 1.804 | 0.838 | 3.884 |
|  | p53 over-expression | 0.097 | 0.580 | 0.305 | 1.103 |
|  | Ki-67 high expression | 0.185 | 0.621 | 0.307 | 1.256 |
|  | Constant | 0.367 | 0.637 |  |  |
| Step 6 | Temporal lobe involvement | 0.481 | 1.264 | 0.658 | 2.430 |
|  | GTR | 0.376 | 0.754 | 0.404 | 1.408 |
|  | IDH1 Mutation | 0.121 | 1.834 | 0.852 | 3.949 |
|  | p53 over-expression | 0.096 | 0.579 | 0.305 | 1.102 |
|  | Ki-67 high expression | 0.166 | 0.608 | 0.301 | 1.229 |
|  | Constant | 0.242 | 0.573 |  |  |
| Step 7 | GTR | 0.371 | 0.752 | 0.403 | 1.404 |
|  | IDH1 Mutation | 0.141 | 1.768 | 0.828 | 3.775 |
|  | p53 over-expression | 0.110 | 0.594 | 0.314 | 1.124 |
|  | Ki-67 high expression | 0.185 | 0.622 | 0.308 | 1.254 |
|  | Constant | 0.313 | 0.630 |  |  |
| Step 8 | IDH1 Mutation | 0.139 | 1.768 | 0.831 | 3.760 |
|  | p53 over-expression | 0.122 | 0.606 | 0.321 | 1.143 |
|  | Ki-67 high expression | 0.181 | 0.621 | 0.309 | 1.248 |
|  | Constant | 0.146 | 0.541 |  |  |
| Step 9 | IDH1 Mutation | 0.026 | 2.185 | 1.097 | 4.352 |
|  | p53 over-expression | 0.127 | 0.612 | 0.325 | 1.150 |
|  | Constant | 0.004 | 0.381 |  |  |
| Step 10 | IDH1 Mutation | 0.035 | 2.075 | 1.051 | 4.098 |
|  | Constant | 0.000 | 0.294 |  |  |

OR, odds ratio; CI, confidence interval; Yrs, years; GTR, gross total resection; IDH1, isocitrate dehydrogenase 1.
